# Stent Thrombosis: Incidence, Predictors and New Technologies

**DOI:** 10.1155/2012/956962

**Published:** 2012-03-11

**Authors:** Gill Louise Buchanan, Sandeep Basavarajaiah, Alaide Chieffo

**Affiliations:** Interventional Cardiology Unit, San Raffaele Scientific Institute, Via Olgettina 60, 20132 Milan, Italy

## Abstract

Some concerns have been raised regarding the risk of late and very late stent thrombosis (ST) following drug-eluting stent implantation. Despite remaining an uncommon complication of percutaneous coronary intervention, when ST occurs, it can be catastrophic to the individual, commonly presenting as acute ST elevation myocardial infarction or sudden cardiac death. The incidence and predictors of ST have been reported in the literature and the role of dual antiplatelet therapies in the avoidance of such a complication remains vital. Ongoing studies are assessing the role of these therapies including platelet reactivity testing, genetic testing and optimum duration of therapy. In addition, newer polymer-free and bioabsorbable stents are under investigation in the quest to potentially minimise the risk of ST.

## 1. Introduction

Drug-eluting stents (DES) were introduced into clinical practice in order to reduce the rates of restenosis observed with bare metal stents (BMS) for the treatment of coronary artery disease [1–6]. However, despite the promising results, some concerns have been raised regarding the potential increased risk of late and very late stent thrombosis (ST) following DES implantation. This is felt to be a consequence of delayed endothelialization [7, 8]. The problem was first highlighted during the 2006 European Society of Cardiology and World Congress of Cardiology Meeting in Barcelona, Spain, when the results of two independent meta-analyses were presented demonstrating a higher mortality with DES [9]. This rapidly became known as the “ESC Firestorm,” and, following this, an abundance of data from both randomized clinical trials and multicenter registries have suggested an increased risk of late and very late ST with the use of DES.

ST is a rare but usually catastrophic event, leading to acute vessel closure, frequently associated with ST elevation myocardial infarction (STEMI) or sudden cardiac death. This paper aims to review the current data regarding ST, the underlying causes and methods to reduce the risk. In addition, there is an emphasis on the importance of dual antiplatelet therapy (DAPT) and newer technologies under development, which may in time lead to a vast reduction in the incidence of ST.

## 2. Definition of Stent Thrombosis according to the Academic Research Consortium

In an attempt to standardize the definition of ST, the Academic Research Consortium (ARC) was formed in 2007, proposing the criteria illustrated in Table 1 [10]. Additionally, the timing of ST can be classified as acute (<24 hours post procedure), early (24 hours to 30 days post procedure), late (31 days to one year post procedure) and very late (>one year post procedure).

## 3. Incidence of Stent Thrombosis

The actual incidence of ST reported in the literature depends on the duration of follow-up utilized. During the early BMS era, ST was demonstrated to be as high as 20.0% [11]. Following this, advances in the field of interventional cardiology, including high pressure post dilatation and importantly, the addition of ticlopidine to aspirin, led to a significant decrease in the occurrence of ST [12, 13]. It was observed that ST in BMS was uncommon after 30 days, due to complete endothelialization within this time [14], which was supported by a pooled analysis of a multicenter trial, when over 80.0% of angiographically confirmed ST occurred within the first 2 days of the procedure [15].

Similarly, most cases of ST occurring with DES occur within 30 days of the procedure. In the Dutch ST registry of over 21,000 patients who were treated with BMS and DES, the majority of ST cases (>70.0%) were seen within the first month, with a cumulative incidence of definite ST of 2.1% over 3 years [16]. There were no differences demonstrated in the incidence of ST between BMS and DES. A further study by the Bern-Rotterdam group, which assessed 8,146 patients treated with DES (paclitaxel-eluting stents (PES) and sirolimus-eluting stents (SES)), reported a cumulative incidence of ST of 3.3% at 3 years. Moreover, the annual incidence of ST in this study was 0.5% [17]. Conversely, a study by a Japanese group (the J-Cypher registry) reported a lower incidence following SES implantation of definite ST at 0.8% over 2 years follow-up [18]. Furthermore, a comprehensive meta-analysis including 9,471 patients from 22 randomized trials, demonstrated no differences in overall mortality (Hazard Ratio (HR) 0.97; 95% Confidence Interval (CI) 0.81–1.15; *P* = 0.72) or myocardial infarction (MI) (HR 0.95; 95% CI 0.79–1.13; *P* = 0.54) with DES compared with BMS. In the observational studies arm of this paper, amongst 182,901 patients in 34 studies, there were significant reductions in mortality (HR 0.78; 95% CI 0.71–0.86) and MI (HR 0.87; 95% CI 0.78–0.97) with DES [19]. A further meta-analysis of 13 randomized trials (7,352 patients) demonstrated that there was no increase in ST with DES (RR 0.97; 95% CI 0.73–1.28) over 2 years [20].

Most of the earlier data in DES is obtained from the first generation DES, with the newer second generation DES thought to pose less risk. Studies evaluating their effectiveness over first generation DES have reported few cases of ST.  Table 2 illustrates the incidence of ST in clinical trials of second generation DES. The trial “Clinical Evaluation of the Xience V Everolimus Eluting Coronary Stent System in the Treatment of Subjects with de Novo Coronary Artery Lesions” (SPIRIT IV), which evaluated the results of everolimus-eluting stents (EES) versus PES, reported ST in only 0.3% of EES patients in the first year compared with 0.8% of PES patients [21]. In the trial “A trial of Everolimus-Eluting Stents and Paclitaxel-Eluting Stents for Coronary Revascularization in Daily Practice” (COMPARE) which randomized 1,800 patients to EES or PES, the incidence of ST at one year was 1.0% following EES as compared with 3.0% in the PES group [22]. In addition, longer-term follow-up to 3 years showed no statistical difference in the occurrence of ST (EES 1.2% versus SES 1.7%) [23]. Similarly, the ENDEAVOR trials which evaluated the Zotarolimus-eluting stent (ZES) reported a significantly lower incidence of ST compared to first generation DES [24–28]. Furthermore, the RESOLUTE all-comers trial showed the rate of ST was lower with EES compared with ZES (0.3% versus 1.2%; *P* = 0.01) [29]. Additionally, a multicenter registry of 4,768 patients has been reported, which specifically assessed ST of second generation DES (EES and ZES). This demonstrated a cumulative incidence of definite ST in 1.0% of patients at 2 years follow-up. The incremental rate of ST from the first year to the second year was 0.25% [30]. Recently, data has shown no occurrence of very late ST at 3 years in 102 patients undergoing primary angioplasty for STEMI [31].

## 4. Class Effect

From the available data, it may be inferred that there is a class effect between the different drugs eluted from the permanent polymer stents. In a meta-analysis of 18,023 patients from randomized trials, there was an increased risk of definite late ST with PES versus SES (HR 1.85; 95% CI 1.02–3.85; *P* = 0.041) [7], which was also shown in other registry studies [34, 35]. Conversely, the “Sirolimus Eluting Stent compared with Paclitaxel Eluting Stent for Coronary Revascularization” (SIRTAX) trial showed no difference between SES and PES (resp., 4.6% versus 4.1%; *P* = 0.74) at 5 years follow-up [36]. Again, although ST is thought to be lower with the second generation “limus” family of drugs than PES [21, 22], no randomized studies have been adequately powered to assess ST; therefore, further data in this field is awaited.

## 5. Prognosis Following Stent Thrombosis

Many patients with ST present either as sudden cardiac death or as an acute STEMI. Those individuals who survive the initial event are known to have a poor prognosis [37]. It is important to emphasize that the case fatality rate in those individuals suffering a ST has been demonstrated to be as high as 45.0% in some series [8, 15, 16, 38–40]. A study of 431 patients with angiographically confirmed definite ST demonstrated almost one in 5 patients with one definite ST experienced another ST at follow-up, showing the high-risk nature following the initial event [16].

Furthermore, a study of 985 patients who underwent primary angioplasty for STEMI, which included 102 patients with definite ST, demonstrated a higher occurrence of in-hospital death or recurrent MI in those presenting with ST rather than STEMI secondary to a de novo lesion (12.7% versus 7.4%; *P* = 0.05) [41]. However, of note, these patients had a higher proportion of comorbidities, including diabetes mellitus, chronic kidney disease and lower left ventricular ejection fraction which may have contributed to the difference in outcomes. Additionally, in those with STEMI secondary to ST, there was a larger thrombus burden, more frequent distal embolization and less successful results from PCI [16, 42].

## 6. Predictors of Stent Thrombosis

In general, ST occurs more frequently in complex patients with complex lesions, for example, acute coronary syndromes, diabetes mellitus, chronic kidney disease, small vessels and multiple stents, including bifurcation lesions and chronic total occlusion [8, 43, 44]. A summary of the underlying patient, lesion and procedural characteristics which are predictors of ST are illustrated in Table 3.

**Figure 1:**
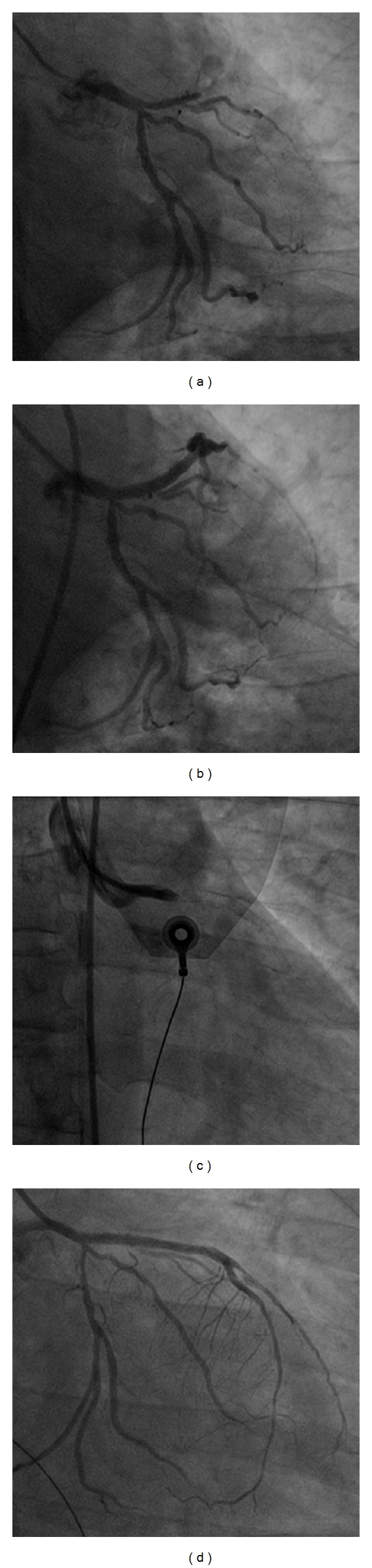
An illustration of how catastrophic stent thrombosis can be when it occurs. Panel (a) shows a chronic total occlusion of the left anterior descending coronary artery which was successfully opened (Panel (b)) with the implantation of everolimus-eluting stents (resp., 3.5 × 33 mm and 2.75 × 33 mm). Twenty-four hours following the procedure, the patient became markedly hypotensive and symptomatic for angina, with EKG showing ST elevation in the anterior leads. Panel (c) demonstrates an acute stent thrombosis at the ostium of the vessel. Finally, Panel (d) shows the results following thrombus aspiration and plain optimal balloon angioplasty.

**Figure 2:**
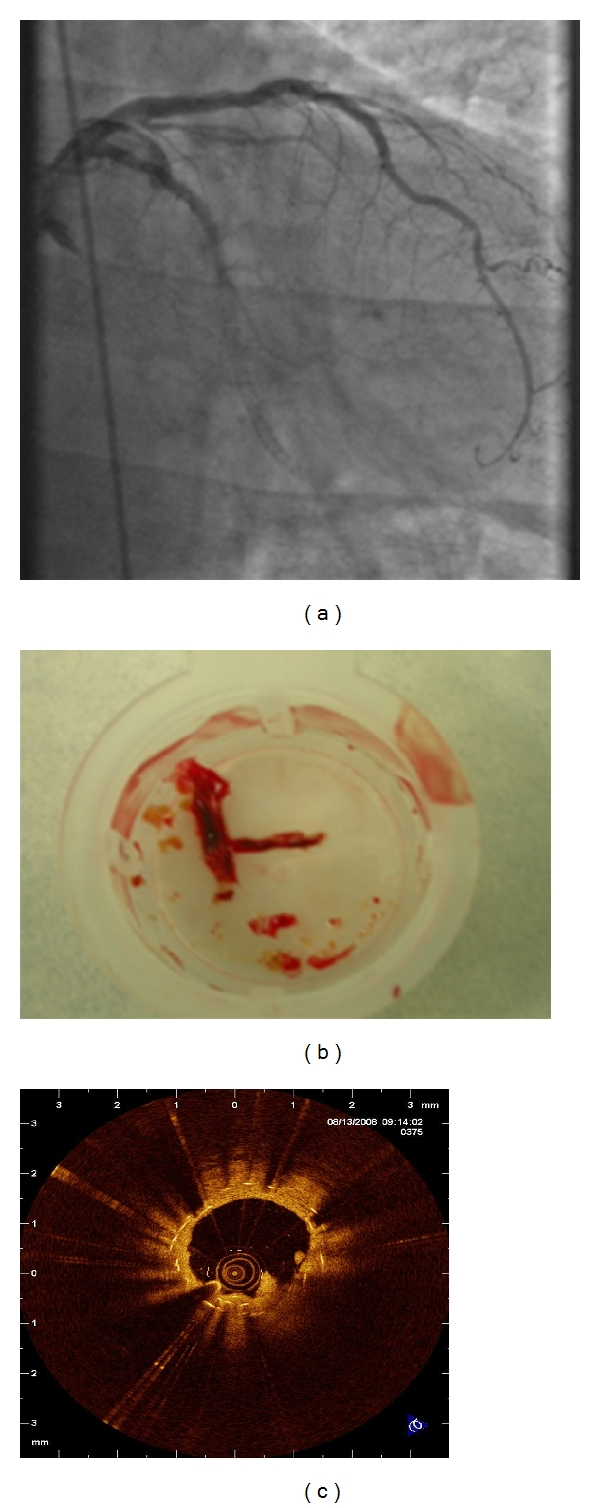
An illustration of a very late stent thrombosis 18 months following Sirolimus-eluting stent implantation in the circumflex artery. Panel (a) demonstrates the angiographic image. Panel (b) shows evidence of thrombus removed with a thrombus extraction system, and, finally Panel (c) illustrates malapposed struts and adherent thrombus following predilatation and assessment with optical coherence tomography.

When a ST develops acutely, it is generally due to procedural-related factors, such as incomplete stent expansion, residual edge dissection, the presence of thrombus and reduced TIMI (Thrombolysis In Myocardial Infarction) flow grade [8, 16, 17, 45].  Figure 1 demonstrates a case of acute ST which occurred in a patient following EES for a chronic total occlusion. The commonest reason for subacute and late ST is the discontinuation of DAPT, which will be discussed in more detail in a later section. Notably, risk factors for the development of very late ST are not so well defined. One possible explanation for such late occurrence is incomplete neointimal coverage as a result of delayed arterial healing, ongoing vessel wall inflammation and late acquired stent malapposition [46–50]. These phenomena have been observed in patients with DES by real-time imaging studies (angioscopy and optical coherence tomography (OCT)) [47, 49–51] and also in some autopsy studies of stented segments in patients with very late ST [46].  Figure 2 demonstrates a definite ST in a patient 18 months following SES implantation and evidence of thrombus on malapposed struts.

## 7. Procedural Optimization

In view of the clinical course associated with a ST, meticulous attention must be made to reduce the risk from the outset. Risk factors pertaining to the patients' history and lesion characteristics are non-modifiable; however, the procedure can be performed optimally to reduce the incidence of ST. Firstly, it is important to adequately screen the patient, to assess likely adherence to the necessary DAPT regimen, the bleeding risk and the need for any planned surgical procedures in the following 12 months.

The decision then needs to be made regarding the anticoagulation of choice during the procedure. However, this also carries risk, as bleeding complications may not be insignificant. Conventionally, unfractionated heparin has been the anticoagulant of choice in those undergoing PCI, often in combination with a glycoprotein IIb/IIIa inhibitor. In the “Controlled Abciximab and Device Investigation to Lower Late Angioplasty Complications” (CADILLAC) trial [52], abciximab use was an independent predictor of no ST (HR 0.27; 95% CI 0.09–0.86; *P* = 0.026). However, recently bivalirudin, a direct thrombin inhibitor, has become more widespread in use. In the “Harmonizing Outcomes with Revascularization and Stents in Acute Myocardial Infarction” (HORIZONS-AMI) trial which randomised 3,602 patients with STEMI undergoing primary angioplasty to heparin plus glycoprotein IIb/IIIa inhibitor versus bivalirudin monotherapy, acute ST occurred in patients assigned to bivalirudin more frequently (1.4% versus 0.3%; *P* < 0.001) with conversely ST after 24 hours occurring less frequently (2.8% versus 4.4%, *P* = 0.02). Notably, there was no difference in the cumulative rates of ST at 2 years between the 2 groups (4.3% versus 4.6%; *P* = 0.73) [53].

Furthermore, good lesion preparation (considering the use of rotational atherectomy in the presence of severely calcified lesions), use of properly sized stents and postdilatation with non compliant balloons, according to intravascular ultrasound (IVUS) guidance, may help adequate stent expansion and apposition. The space between the stent struts and vessel wall which is present when there is malapposition leads to an area of sluggish flow, which can allow thrombus formation. It is therefore essential to choose the correct stent size and perform effective high pressure postdilatation to reduce this risk. Adequate stent expansion on IVUS has been linked to lower ST at both 30 days and 12 months [54]. This can also ensure there are no remaining edge dissections which may be a nidus for ST.

 Newer imaging modalities, such as OCT, may become a useful tool to help assessment of the substrates for ST development, thereby allowing optimization of the initial procedure. As discussed earlier, incomplete stent apposition may be associated with the formation of thrombus; on OCT, thrombus was seen significantly more frequently in struts that were not fully apposed compared to those with good apposition (20.6% versus 2.0%; *P* < 0.001) [55]. Additionally, it has been shown that DES have a higher rate of uncovered and malapposed struts than BMS with OCT imaging [56].

## 8. Correlation between Dual Antiplatelet Therapy and Stent Thrombosis

The current recommendations from the American College of Cardiology/American Heart Association/Society for Cardiovascular Angiographic Intervention are that, following DES implantation, patients should receive clopidogrel (or an alternative thienopyridine) in addition to aspirin for a minimum of 12 months, unless there is a high bleeding risk or urgent circumstances arise [57]. The Task Force on myocardial revascularization of the European Society of Cardiology and the European Association for Cardiothoracic Surgery is less specific and state that convincing data exists only for continuation of DAPT up to 6 months [58].

However, the optimal duration after DES implantation remains unknown. The discontinuation of DAPT was the most powerful predictor of ST during the first 6 months following stent implantation in a cohort of over 3,000 patients (HR 13.74; 95% CI 4.04–46.68; *P* < 0.001) [59]. This was confirmed by a study by Schulz et al. in over 6,000 patients with 4-years follow up [60]. Furthermore, it has been demonstrated that ST occurred in 29.0% of patients who prematurely discontinued DAPT [8]. Conversely, the Bern/Rotterdam registry of over 8,000 patients demonstrated no differences in those treated with clopidogrel for 12 months following DES implantation compared with those treated for only 3–6 months.

Alternatively, it has been suggested that patients treated with clopidogrel for 24 months had improved cumulative survival compared to those treated for 12 months [61]. However, it has been shown in a randomized trial of 2,701 patients who were free of major adverse cardiovascular and cerebrovascular events (MACCE) and major bleeding events for 12 months alter PCI receiving 12 versus 24 months of clopidogrel that the cumulative occurrence of definite ST at 24 months after DES was identical in patients who continued clopidogrel until 24 months (0.4% versus 0.4%; *P* = 0.76) [62]. The “PROlonging Dual-antiplatelet treatment after Grading stent-Induced hyperplasia” [63] (PRODIGY) study was presented at the European Society of Cardiology Scientific Congress, Paris, 2011 which randomized 2,013 patients on an intention-to-stent basis to EES (*n* = 501), PES (*n* = 505), ZES (*n* = 502), or BMS (*n* = 505). After 30 days, 1,970 were eligible for randomization to either 6 months DAPT (*n* = 983) or 24 months DAPT (*n* = 987) and followed up for 2 years. This showed no benefit of prolonged DAPT in the incidence of ischemic events at the cost of an increased risk of major bleeding (HR 2.17; 95% CI 1.44–3.22; *P* = 0.00018). The optimal duration is currently being assessed in a number of randomized trials illustrated in Table 4.

 A number of factors have been thought to be implicated in the development of ST including clopidogrel resistance, hypersensitivity to the stent polymer, drug interactions and the discontinuation of clopidogrel within the first 6 months [59]. The measurement of platelet function on DAPT can enable those patients at highest risk to be identified [66–68], as it is known individual responses to clopidogrel vary significantly [69]. One quarter of individuals may be resistant to the platelet-inhibiting effects of clopidogrel [70, 71]. A recent meta-analysis of over 3,000 patients demonstrated that high on treatment platelet reactivity tested by the VerifyNow assay (Accumetrics, San Diego, California, USA), defined as a P2YC12 Reaction Unit (PRU) of greater than or equal to 230, was associated with ST (HR 3.11; 95% CI 1.50–6.46; *P* = 0.002) [72]. Moreover, Parodi et al. demonstrated in 1,789 patients undergoing PCI who had platelet reactivity prospectively assessed using light transmittance aggregometry that a high residual platelet reactivity (≥70% platelet aggregation) led to a higher incidence of ST (6.1% versus 2.9%; *P* = 0.01) [73]. However, even if we are aware of this, no benefit was shown in the “Gauging Responsiveness With a VerifyNow Assay: Impact on Thrombosis and Safety” (GRAVITAS) trial following double dose clopidogrel if there was high on treatment platelet reactivity [74]. In this study, the composite rate of cardiovascular death, MI, or ST was low in both groups (2.3%) at 6 months. However, it is important to note that less than half of patients randomized to clopidogrel 150 mg achieved appropriate variability in antiplatelet response, which may have contributed to the lack of observed clinical effect. In addition, due to the lower than expected event rates, the study was underpowered to detect the clinical efficacy of high-dose clopidogrel. Another randomized study, “Testing Platelet Reactivity in Patients Undergoing Elective Stent Placement on Clopidogrel to Guide Alternative Therapy With Prasugrel” (TRIGGER-PCI), comparing prasugrel to clopidogrel for those with high platelet reactivity was terminated early due to futility as high on clopidogrel platelet reactivity (>208 PRU by VerifyNow) was observed less frequently than expected and there was a low occurrence of the primary endpoint of cardiac death or MI. There are a number of ongoing studies which aim to assess the utilization of platelet reactivity testing and optimization of DAPT following PCI, which are illustrated in Table 5.

Clopidogrel is a prodrug which requires conversion to an active metabolite to provide its effects and resistance can be due to a genetic variation in one of the cytochrome P450 hepatic enzymes necessary for this, particularly the CYP2C19 allele [75]. Patients with polymorphisms in this gene, which accounts for only 5–12% of clopidogrel response variability [76, 77], have more adverse clinical events following PCI. [78, 79]. Indeed, this gene has been independently shown to be associated with early ST [80]. Despite this, currently genetic testing is not widely utilized in patients requiring stent implantation; however, the “Escalating Clopidogrel by Involving a Genetic Strategy—Thrombolysis In Myocardial Infarction 56” (ELEVATE-TIMI) study was recently reported [81]. The aim of this study was to assess whether higher doses of clopidogrel (up to 300 mg) improved responses in the setting of loss of function CYP2C19 genotypes. The genotypes of 333 patients with stable coronary artery disease were established, and it was shown that a dose of 225 mg clopidogrel daily in CYP2C19 heterozygotes achieved levels of platelet reactivity similar to that seen with the standard 75 mg dose in non-carriers. However, in homozygotes, doses of even 300 mg did not result in comparable degrees of platelet inhibition. A number of studies are currently ongoing to further assess the role of CYP2C19 pharmacogenomics (Table 6). Notably, the US Food and Drug Administration has added a box warning to clopidogrel about the reduced effectiveness in those patients who are poor metabolizers of the drug.

 If a patient develops ST while fully compliant with DAPT, then consideration should be given to the modification of the drug regimen. Newer antiplatelet agents, including prasugrel [82] and ticagrelor [83], are now available which have been shown to be more potent, with a consequent reduction in ST [84, 85]. Non responders to clopidogrel also respond well to these newer agents [86, 87]. In patients with acute coronary syndromes, the rates of ST have been reduced by substituting clopidogrel with these drugs, at the risk of increased bleeding [88, 89]. There is also the option of adding in therapy such as cilostazol, a phosphodiesterase inhibitor [90]. This has been shown in a registry study of 3,099 patients to reduce the risk of ST at 12 months as compared to DAPT only (HR 0.136; 95% CI 0.035–0.521; *P* = 0.0036) [91], with no increase in bleeding complications. However, most studies of cilostazol have been performed in Asians and have not been validated in the Western population.

Finally, drug interactions from other hepatic enzyme inhibitors can result in reduced effectiveness of clopidogrel. Importantly, in recent years, there has been concern regarding the use of concomitant proton pump inhibitors (PPIs) with clopidogrel [92]. However, the “Clopidogrel with or without omeprazole in coronary artery disease” (COGENT) trial which randomly assigned 3,873 patients on DAPT to omeprazole or placebo demonstrated no apparent cardiovascular interaction between the drugs [93]. However, this study was terminated early and therefore was underpowered for the endpoint. The American Heart Association guidelines do not prohibit the use of PPIs, yet highlight the potential risks and benefits of coadministering with clopidogrel [94]. Ongoing studies are assessing the role of CYP2C19 in the drug interaction between clopidogrel and PPIs: the “Evaluation of the Influence of Statins and Proton Pump Inhibitors on Clopidogrel Antiplatelet Effects” (SPICE) trial and the Influence of CYP2C19 Genetic Variants on Clopidogrel in Healthy Subjects Study.

## 9. Impact of New Technologies

Currently, there is much interest in the development of new technologies to improve safety outcomes, including a reduction in ST. It has been known for a number of years that durable polymers can cause local arterial injury [95]. Non erodible polymers provoke chronic eosinophilic infiltration within the arterial wall, suggestive of hypersensitivity reactions [96, 97]. Concerns regarding the effects of these polymers had led to the design of stents with either biodegradable polymers or indeed no polymers, which are illustrated in Table 7.

 The “Limus Eluted from A Durable versus ERodable Stent coating” (LEADERS) trial randomized 1,707 patients to either SES or biolimus A9-eluting stents (BES). The latter stent elutes biolimus from a polylactide biodegradable polymer applied to the abluminal surface of the stent which is fully metabolized within 6–9 months. At 3 years follow up, the rate of definite ST was 2.2% for the BES and 2.9% for the SES (HR 0.78; 95% CI 0.43–1.43; *P* = 0.43). However, interestingly, ST increased at a lower rate of 0.2% from one to 3 years for the BES compared with 0.9% for the SES. In addition, patients who had stopped DAPT did not appear to have any additional events at 3 years, with conversely in the SES group, there were development of events [98]. Furthermore, this may be explained by an OCT substudy of the LEADERS trial which demonstrated that BES had better strut coverage at 9 months than SES [99].

 Further stents with biodegradable polymers are being developed and are showing promising results in early trials. The Biofreedom stent (Biosensors, Morges, Switzerland), which again is a BES, has demonstrated minimal delayed arterial healing [100]. Additionally, the Synergy stent (Boston Scientific, Natick, MA, USA) which has a bioabsorbable polymer and everolimus drug combination has recruited patients to the EVOLVE study (non-inferiority trial to assess the safety and performance of the evolution coronary stent), and the results are awaited. Finally, the CRE8 (CID, Saluggia, Italy) stent has recently gained CE mark. This is a unique polymer free abluminal reservoir technology together with a new amphilimus formulation which allows controlled and targeted drug elution to the artery. The results of the “International Randomized Comparison between DES Limus Carbostent and Taxus Drug Eluting Stents in the Treatment of De Novo Coronary Lesions” (NEXT) trial were presented at EuroPCR, Paris, France, in 2011 which demonstrated no ST at 180 days.

 More recently, completely bioabsorbable stents have been developed to allow complete stent resorption, arterial healing and restoration of normal vascular function. As stent struts theoretically disappear, issues related to late persistent strut malapposition and chronically uncovered struts, including ST, become irrelevant. Additionally, there is elimination of chronic sources of vessel irritation and inflammation which could potentially reduce the need for prolonged DAPT. The “Clinical Evaluation of the Bioabsorbable Vascular Solutions Everolimus Eluting Coronary Stent System in the Treatment of Patients with Single De Novo Coronary Artery Lesions” (ABSORB) trial was a prospective multicenter, open-label, first-in-man study that assessed the bioabsorbable polymer stent in 30 patients. This has shown no cases of ST at 4 years follow up [101]. The results of the second cohort of the ABSORB trial with the second generation bioabsorbable stent have demonstrated no cardiovascular death at 12 months follow-up [102]. Currently, patients are being recruited for ABSORB Extend which aims to recruit 1,000 patients and follow up for the occurrence of ST. There are several bioabsorbable stents in development and in clinical trials. The REVA stent (REVA medical Inc., San Diego, CA, USA) coated with paclitaxel and the Bioabsorbable Therapeutics stent (Bioabsorbable Therapeutics Inc., Menlo Park, CA, USA) coated with sirolimus are currently being tested.

## 10. Conclusions

The occurrence of a ST is rare, however, remains one of the most feared complications following PCI, due to the potential catastrophic consequences. There are a number of factors that may lead to the development of a ST and it is essential that we optimize PCI technique and importantly DAPT regimens in all patients undergoing PCI. Newer stents are being developed at rapid rates, and future large-scale randomized trials may help in the decision making regarding these issues in an aim to reduce this dramatic event.

## Figures and Tables

**Table 1:** ARC criteria for the diagnosis of stent thrombosis.

Definite	Angiographic or pathologic evidence of ST
Probable	Unexplained death within 30 days of the procedure or MI at any time in the territory of previous PCI
Possible	Unexplained death occurring 30 days post procedure

ST: stent thrombosis; MI: myocardial infarction; PCI: percutaneous coronary intervention.

**Table 2:** Studies of second generation drug-eluting stents and the incidence of stent thrombosis.

Study	Stent types	Followup (months)	Incidence of ST (%)
SORT OUT III [32]	ZES versus SES	18	0.5 versus 1.0
Resolute all-comers [29]	ZES versus EES	12	1.2 versus 0.3
ZEST [33]	ZES versus SES versus PES	12	0.7 versus 0 versus 0.8
ENDEAVOR IV [24]	ZES versus PES	12	0.7 versus 0.1
SPIRIT IV [21]	EES versus PES	12	0.3 versus 0.8
COMPARE [22]	EES versus PES	12	1.0 versus 3.0

SORT OUT III: Randomized Comparison of the Endeavor and the Cypher Coronary Stents in Non-Selected Angina Pectoris Patients; ZEST: Comparison of the Efficacy and the Safety of Zotarolimus-Eluting Stent Versus Sirolimus-Eluting Stent and PacliTaxel-Eluting Stent for Coronary Lesions; SPIRIT IV: Clinical Evaluation of the Xience V Everolimus Eluting Coronary Stent System in the Treatment of Subjects with de Novo Coronary Artery Lesions; COMPARE: A Trial of Everolimus-Eluting Stents and Paclitaxel-Eluting Stents for Coronary revascularization in Daily Practice; SES: Sirolimus-Eluting stent; PES: Paclitaxel-eluting stent; ZES: Zotarolimus-eluting stent; EES: Everolimus-eluting stent.

**Table 3:** Predictors of stent thrombosis.

Patient characteristics	Lesion characteristics	Procedural characteristics
Diabetes mellitus	Long segment of disease	Stent underexpansion
Chronic kidney disease	Small diameter vessel	Stent malapposition
Acute presentation	Saphenous venous graft	Edge dissection
Current smoker	Chronic total occlusion	Strut fracture
Reduced left ventricular function	Bifurcation lesion	Multiple stent implantation and stent overlap
Cancer		Geographic miss and residual stenosis
DAPT non-responsiveness		Reduced TIMI flow alter procedure
Premature cessation of DAPT		
Advanced age		
Thrombocythemia		
Hypersensitivity to polymer or drug		

DAPT: dual antiplatelet therapy; TIMI: thrombolysis in myocardial infarction.

**Table 4:** Studies of ongoing randomized trials to assess optimal clopidogrel duration.

Study	Patient population	Clopidogrel duration (months)	Primary endpoint
ISAR-SAFE [64]	Patients on clopidogrel 6 months alter DES	6 versus 12	Composite of death, MI, ST, stroke and major bleeding
DAPT [65]	All-comers	12 versus 30	Composite of death, MI, SI, stroke and major bleeding
DAPT-STEMI	All STEMI patients	6 versus 12	MACCE
SECURITY	Second-generation DES	6 versus 12	Definite/probable ST
RESET	All-comers	3	Composite of cardiovascular death, MI, ST and major bleeding
OPTIMIZE	Stable CAD and NSTEMI	3 versus 12	Composite of death, MI, stroke and major bleeding

ISAR-SAFE: Intracoronary Stenting and Antithrombotic Regimen: Safety and Efficacy of 6 Months Dual Antiplatelet Therapy After Drug-Eluting Stenting; DAPT: Dual Antiplatelet Therapy Study; STEMI: ST Elevation Myocardial Infarction; SECURITY: SECond generation drUg-eluting stents implantation followed by six-versus twelve-month dual antIplatElet therapy; RESET: A New Strategy Regarding Discontinuation of Dual Antiplatelets; OPTIMIZE: Optimized Duration of Clopidogrel Therapy Following Treatment with the Endeavor Zotarolimus Eluting Stent in the Real World Clinical Practice; DES: drug-eluting stent; CAD: coronary artery disease; NSTEMI: non-ST elevation myocardial infarction; MI: myocardial infarction; ST: stent thrombosis; MACCE: major adverse cardiovascular and cerebrovascular events.

**Table 5:** A table to illustrate ongoing studies utilizing the results of platelet reactivity testing to assess outcomes following PCI.

Study	Number of patients	Patient population	Platelet reactivity value	Antiplatelet therapy	Primary endpoint
ARCTIC	2,500	Elective PCI	<15%	GPIIb/IIIa inhibitors	Death, MI, Stroke, TVR, ST
DANTE	442	NSTEMI	PRU > 240	Clopidogrel 75 mg versus Clopidogrel 150 mg	Cardiovascular death, MI and TVR

ARCTIC: Monitored Adjusted Antiplatelet Treatment Versus a Common Antiplatelet Treatment for DES Implantation and Interruption Versus Continuation of Double Antiplatelet Therapy; DANTE: Dual Antiplatelet Therapy Tailored on the Extent of Platelet Inhibition; PCI: percutaneous coronary intervention; NSTEMI: Non-ST elevation myocardial infarction; GPIIb/IIIa: Glycoprotein IIb/IIIa inhibitor; MI: myocardial infarction; TVR: target vessel revascularization; ST: stent thrombosis; PRU: P2YC12 reaction unit.

**Table 6:** Ongoing studies assessing pharmacogenomics and antiplatelet therapy.

Study	Number of patients	Patient population	Therapy	Primary endpoint
GIFT	NA	All-comers undergoing PCI	Tailored clopidogrel versus standard clopidogrel according to platelet reactivity and genetic type	Residual platelet activity
TARGET-PCI	1,500	Nonemergent PCI	Tailored with clopidogrel and prasugrel to results of platelet reactivity and genetic type	MACE
CLOVIS-2	120	Post-MI	Clopidogrel 300 mg versus Clopidogrel 600 mg in 2 genetic CYP2C19 types	Inhibition of residual platelet activity 6 hours following clopidogrel
PREDICT	42	Stable CAD	Those with high residual platelet activity on clopidogrel and genotyped for CYP2C19 treated with double dose clopidogrel	Change in residual platelet activity
GeCCO	14,600	Recent ACS	Genotype-guided comparison of clopidogrel in extensive metabolizers and prasugrel	Cardiovascular death, nonfatal MI, or nonfatal stroke

GIFT: Genotype Information and Functional Testing; TARGET-PCI: Thrombocyte Activity Reassessment and Genotyping for PCI; CLOVIS-2: Clopidogrel and response Variability Investigation Study 2; PREDICT: Pilot Study on the Effect of High Clopidogrel Maintenance Dosing; GeCCO: Genotype Guided Comparison of Clopidogrel and Prasugrel Outcomes Study; PCI: percutaneous coronary intervention; MI: myocardial infarction; CAD: coronary artery disease; ACS: acute coronary syndrome; MACE: major adverse cardiovascular event.

**Table 7:** Newer stents.

Stent	Stent Platform	Polymer	Drug	Company
Biomatrix	Stainless steel	PLA	Biolimus-A9	Biosensors Inc., Newport Beach, CA, USA
JACTAX	Stainless steel	PLA	Paclitaxel	Boston Scientific, Natick, MA, USA
Nobori	Stainless steel	PLA	Biolimus-A9	Termumo Medical Corp., Tokyo, Japan
Synergy	Platinum chromium	PLA	Everolimus	Boston Scientific, Natick, MA, USA
Janus Flex	Stainless steel	Carbofilm	Tacrolimus	Sorin, Italy
Biofreedom	Stainless steel	None	Biolimus-A9	Biosensors Inc., Newport Beach, CA, USA
Cre8	Cobalt chromium	None	Amphilimus	CID, Saluggia, Italy
Amazonia Pax	Cobalt chromium	None	Paclitaxel	Minvasys, Paris, France
VESTAsync	Stainless steel	None	Sirolimus	MIV Therapeutics, Atlanta, GA, USA
Yukon Choice	Stainless steel	None	Sirolimus	Translumina, Hechingen, Germany

PLA: polylactic acid.
